# More oxygen during development enhanced flight performance but not thermal tolerance of *Drosophila melanogaster*

**DOI:** 10.1371/journal.pone.0177827

**Published:** 2017-05-23

**Authors:** Shayan Shiehzadegan, Jacqueline Le Vinh Thuy, Natalia Szabla, Michael J. Angilletta, John M. VandenBrooks

**Affiliations:** 1 School of Life Science, Arizona State University, Tempe, Arizona, United States of America; 2 Institute of Environmental Studies, Jagiellonian University, Kraków, Poland; 3 Department of Physiology, Midwestern University, Glendale, Arizona, United States of America; Clemson University, UNITED STATES

## Abstract

High temperatures can stress animals by raising the oxygen demand above the oxygen supply. Consequently, animals under hypoxia could be more sensitive to heating than those exposed to normoxia. Although support for this model has been limited to aquatic animals, oxygen supply might limit the heat tolerance of terrestrial animals during energetically demanding activities. We evaluated this model by studying the flight performance and heat tolerance of flies (*Drosophila melanogaster*) acclimated and tested at different concentrations of oxygen (12%, 21%, and 31%). We expected that flies raised at hypoxia would develop into adults that were more likely to fly under hypoxia than would flies raised at normoxia or hyperoxia. We also expected flies to benefit from greater oxygen supply during testing. These effects should have been most pronounced at high temperatures, which impair locomotor performance. Contrary to our expectations, we found little evidence that flies raised at hypoxia flew better when tested at hypoxia or tolerated extreme heat better than did flies raised at normoxia or hyperoxia. Instead, flies raised at higher oxygen levels performed better at all body temperatures and oxygen concentrations. Moreover, oxygen supply during testing had the greatest effect on flight performance at low temperature, rather than high temperature. Our results poorly support the hypothesis that oxygen supply limits performance at high temperatures, but do support the idea that hyperoxia during development improves performance of flies later in life.

## Introduction

Despite decades of research, the question of what causes animals to die at high temperatures remains unresolved. Several mechanisms have been proposed, including the destabilization of proteins or membranes and the failure of cell signaling [1–4]. A critical flaw in these proposals is that animals exposed to thermal stress often die at temperatures well below those predicted by these mechanisms [5]. To address this problem, Pörtner proposed a model in which animals fail at high temperatures because oxygen demand exceeds the supply [5–7]. This model assumes that high temperatures require animals to rely on anaerobic metabolism, which provides insufficient energy and thus impairs the function of nerves and myocytes. If Pörtner’s model is correct, an animal’s susceptibility to hypoxia and its ability to acquire oxygen should determine its heat tolerance [8, 9]. Without a compensatory change in the respiratory and cardiovascular system, a decreasing supply of oxygen in an environment should ultimately make an animal more susceptible to overheating.

Studies of aquatic animals tend to support the oxygen-limitation hypothesis [9–12], but studies of terrestrial animals have yielded less support. Recently, lizards were found to prefer lower temperatures [13] or lose mobility at lower temperatures when exposed to hypoxia compared to normoxia. However, most terrestrial animals, including lizards, will never experience the extremely low levels of oxygen used in these experiments. Moreover, the vast majority of animals are insects, which possess a sophisticated network of tracheae and tracheoles that deliver oxygen directly to mitochondria [14]. Obviously, any unifying model of how oxygen supply limits thermal tolerance would need to consider the physiology of insects under realistic levels of oxygen. As oxygen supply decreases, insects can open their spiracles more frequently, ventilate their tracheal system more rapidly, and modulate their fluids to enhance diffusion [15]. At high temperatures, such responses must enable some insects to meet the greater demand for oxygen, because hypoxia did not reduce heat tolerances of dragonflies, cockroaches, or flies in previous experiments [8, 16, 17]. Even in species that tolerated heat worse under hypoxia, hyperoxia failed to enhance heat tolerance, as one might expect [reviewed by 9]. So far, oxygen supply seems to limit heat tolerances of terrestrial animals only during the embryonic stage [18, 19], when the respiratory system is still developing and oxygen delivery depends on diffusion across an eggshell [20].

Despite these results, the generality of Pörtner’s model might have been underestimated because researchers have tested its predictions mainly by studying animals at rest. Resting animals consume far less energy than do active animals. The combined stress of heat and hypoxia should challenge animals most during strenuous activities, such as running or flying. During flight, the muscles of insects demand more oxygen than just about any tissue [21, 22]. In previous experiments, hypoxia reduced the maximal aerobic metabolism of insects [15], and hyperoxia increased aerobic metabolism and flight performance [23]. Thus, the thermal tolerance of insect flight is a logical subject for evaluating the generality of Pörtner’s oxygen limitation hypothesis.

We tested the oxygen limitation hypothesis by quantifying chronic and acute effects of oxygen supply on the flight performance and thermal tolerance of flies (*Drosophila melanogaster*). One of the advantages of working with insects is that it has been shown that their respiratory system can be manipulated during development by altering oxygen supply. Developmental plasticity in the number and size of tracheae has been observed in a variety of insects [24, 25], including *D*. *melanogaster* [26, 27]. This plasticity could potentially enhance the heat tolerance of an adult insect in a hypoxic environment. If this reasoning holds, exposure to hypoxia during larval development should enhance tolerance of hypoxia and heating as an adult. Therefore, we predicted that development at hypoxia would lead to greater tolerance of hypoxia and heat during adulthood. These effects should be stronger under energetically demanding activities, such as flight, than at rest. As a tradeoff, however, a more elaborate network of tracheae could leave an insect more susceptible to oxidative damage or water loss in a normoxic or hyperoxic environment [15]. Additionally, a tradeoff between investing in tracheal systems and other tissues, such as flight muscles and related tissues, would increase the flight performance of insects reared under hyperoxia [15, 28].

## Materials and methods

### Origin and maintenance of isofemale lines

We studied the acclimation of flies from a natural population of *D*. *melanogaster* collected in Danville, Indiana, USA. Isofemale lines derived from this population were raised at 20.5°C and normoxia for five generations before our first experiment. During this time, lines were maintained in 25 x 90 mm vials (Genesee Scientific, San Diego, USA) on ~3–4 cm of the Bloomington Standard corn meal-corn syrup diet. Adults from each line were transferred to fresh vials every 3–4 weeks to minimize overlap between generations. Prior to each experiment, we controlled the density of each isofemale line for two generations by transferring only two adults of each sex into new vials to reproduce for 48 h.

At the beginning of each experiment, pairs of adult flies from each isofemale line were transferred to new vials kept at certain levels of oxygen (see details of each experiment below). Oxygen concentrations were maintained by a commercial oxygen controller (ROXY-8; Sable Systems International, Las Vegas, Nevada, USA). Temperature was maintained at 20.5°C by a programmable incubator (DR-36VL; Percival Scientific, Perry, Iowa, USA). Adults that emerged under these conditions were used in our studies of flight performance and heat tolerance.

### Acclimation of flight performance

We raised flies from six isofemale lines at each of three concentrations of oxygen (12%, 21%, and 31%) and studied their flight performance as adults. Seven to ten days after eclosion, flies were tested at all combinations of three air temperatures (37°C, 39°C, and 41°C) and three oxygen concentrations (12%, 21%, and 31%). Additionally, we tested a subset of flies, raised under normoxia, at 25°C to test whether performances were less sensitive to oxygen supply at a benign temperature. Twenty-four hours before testing, females were anesthetized with CO_2_, transferred to new vials, and returned to an incubator set at 20.5°C. To prevent effects of anesthesia on flight [29], each fly was transferred without anesthesia to an empty vial just before testing.

We quantified flight performances in a custom chamber [30]. The flight chamber (30.5 x 30.5 x 30.5 cm) was constructed from clear acrylic, with a circular opening at the top. This opening was temporarily sealed by a movable plate. As a vial with a fly was inverted on top of this plate, holes in the plate enabled the temperature and oxygen concentration of the vial to equilibrate with levels in the chamber. A fan inside the box circulated air from a commercial oxygen controller (ROXY-1, Sable Systems International, Las Vegas, Nevada, USA). Preliminary tests showed that temperature and oxygen concentration of the vial matched those of chamber within 3.5 min. After this period, the plate was slid aside and the fly was tapped into the chamber. Flies either fell uncontrollably or flew to a surface. To ensure objectivity, a fall was scored when a fly landed on the floor within 10 cm of the point below the vial. The order of testing at each temperature and oxygen concentration was randomized among developmental treatments and isofemale lines.

### Acclimation of heat tolerance

We raised flies from four isofemale lines at each of eight concentrations of oxygen (10%, 12%, 15%, 18%, 21%, 24%, 27%, and 30%) and measured their heat tolerance as adults (N ≥ 30 for each treatment). Heat tolerance was estimated as the time required for a fly to lose motor control at 39.5°C in a normoxic atmosphere (21%), also known as knockdown time [31]. Each day, newly emerged flies from each isofemale line were isolated in new vials. Between 5 and 9 days after emergence, each fly was transferred without anesthesia into a glass vial (10 mL) with a stopper. Flies were kept in vials for fewer than 5 min before heat tolerance was measured.

We measured knockdown time in vials suspended in a custom water bath. The bath consisted of a clear acrylic box (28 x 4 x 7.5 cm) with a sealed, watertight lid. Eight vials were suspended from holes in the lid. Water flowed into one end of the box and out the other end, such that vials were completely submerged in water when the box was sealed. The temperature of the water was controlled at 39.5°C (± 0.1°C) by a commercial circulator (Model 11505, VWR, USA). This temperature was based on previous studies [32, 33], in which flies succumbed quickly enough to minimize effects of energy or water loss but slowly enough to record the knockdown times precisely. In each trial, the time until a fly collapsed was recorded as the knockdown time. Times were recorded with by the JWatcher computer software [34].

### Statistical analyses

We modeled dependent variables using the lme4 library [35] of the R Statistical Package [36]. For flight performance, we fit a linear mixed model with binomial distribution of error and fixed effects of oxygen during development, oxygen during testing, and temperature during testing. For knockdown time, we used a linear mixed model with a normal distribution of error and fixed effects of sex and oxygen during development. In each analysis, the effect of isofemale line was modeled as a random factor.

Following Burnham and Anderson [37], we used multi-model averaging to estimate the most likely values of means. First, we estimated the most likely random effects according to Zuur and colleagues [38]. Then, we used the MuMIn library [39] to fit all possible sets of fixed effects to the data. Finally, we calculated the Akaike information criterion (*AIC*_*C*_) and Akaike weight of each model (Tables 1 and 2). To calculate the mean for each group, we used the weighted average of each parameter, including estimates from all models. This approach focuses on estimates of effect size and eliminates the need for *P* values, because all models (including the null model) contribute to the most likely value of each mean [37].

**Table 1:** All likely models included an effect of temperature on flight performance. The two most likely models also included an effect of developmental temperature. For each model, we provide the Akaike information criterion (*AIC*_*c*_) and the Akaike weight, which equals the probability that the model describes the data better than other models. All models contained an intercept and an error term associated with isofemale line.

*Model*	*Parameters*	*Log likelihood*	*AIC*_*c*_	*ΔAIC*_*c*_	*Akaikeweight*
**1)** sex + dev[O_2_] + temp	6	-124.0	260.2	0	0.19
**2)** dev[O_2_] + temp	5	-125.2	260.6	0.34	0.16
**3)** sex + temp	4	-126.4	261.0	0.79	0.13
**4)** temp	3	-127.5	261.1	0.85	0.13
**5)** dev[O_2_] + temp + test[O_2_] + (dev[O_2_] ∙ test[O_2_])	11	-3119.9	262.7	2.46	0.06
**6)** dev[O_2_] + temp + test[O_2_] + (dev[O_2_] ∙ test[O_2_]) + (temp ∙ test[O_2_])	15	-115.8	263.2	3.00	0.04
**7)** sex + dev[O_2_] + temp + test[O_2_] + (dev[O_2_] ∙ test[O_2_])	12	-119.2	263.5	3.27	0.04
**8)** sex + dev[O_2_] + temp + test[O_2_]	8	-123.6	263.8	3.52	0.03
**9)** dev[O_2_] + temp + test[O_2_]	7	-124.8	264.0	3.75	0.03
**10)** temp + test[O_2_]	5	-127.1	264.4	4.19	0.02
**11)** sex + temp + test[O_2_]	6	-126.1	264.5	4.22	0.02
**12)** sex + dev[O_2_] + temp + test[O_2_] + (dev[O_2_] ∙ test[O_2_]) + (temp ∙ test[O_2_])	16	-115.4	264.6	4.33	0.02
**13)** dev[O_2_] + temp + test[O_2_] + (temp ∙ test[O_2_])	11	-120.8	264.6	4.34	0.02
**14)** sex + dev[O_2_] + temp + test[O_2_] + (temp ∙ test[O_2_])	12	-119.9	264.8	4.52	0.02
**15)** temp + test[O_2_] + (temp ∙ test[O_2_])	9	-123.3	265.2	5.00	0.02
**16)** dev[O_2_] + temp + (dev[O_2_] ∙ temp)	9	-123.6	265.7	5.44	0.01
**17)** sex + temp + test[O_2_] + (temp ∙ test[O_2_])	10	-122.5	265.7	5.44	0.01

**Table 2:** The most likely model of knockdown time included only an effect of sex. All other models were poorly supported (*AIC*_*c*_ > 6). For each model, we provide the Akaike weight, which equals the probability that the model describes the data better than other models. All models contained an intercept and an error term associated with isofemale line.

*Model*	*Parameters*	*Log likelihood*	*AIC*_*c*_	*ΔAIC*_*c*_	*Akaikeweight*
**1)** sex	4	-648.5	1305.2	0	0.95
**2)** sex + test[O_2_] + (sex ∙ test[O_2_])	18	-636.4	1311.4	6.17	0.04
**3)** sex + test[O_2_]	11	-645.7	1314.4	9.20	0.01

## Results

**Fig 1:**
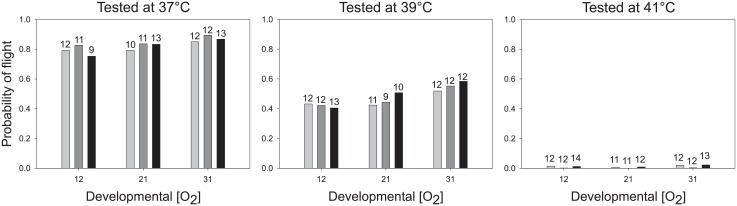
Flight performance depended on body temperature and oxygen supply. At 37°C (left) and 39°C (center), flies performed better if they had developed with a greater supply of oxygen. At 41°C (right), flies performed poorly overall. The color of each bar denotes the oxygen level at which flies were tested (light gray = 12%, dark gray = 21%, black = 31%). The most likely probability of flight under each condition was computed by multimodel averaging. The number of observations used to estimate the mean is marked at the top of each bar.

Flight was affected more by temperature than by oxygen concentration, during development and during testing (Table 3). All likely models, summing to an Akaike weight of 1, included an effect of temperature (see Table 2). When these effects were averaged across models, the probability of flight decreased as temperature increased from 37° to 41°C. This effect was so strong that flies generally flew when tested at 37°C but rarely flew when tested at 41°C (Fig 1).

**Table 3:** The importance of factors in our models of flight performance and knockdown time. Importance equals the sum of Akaike weights for models that include the factor (or the probability that the factor would occur in the best model). A dash indicates that a factor was not considered in the set of models.

*Factor*	*Importance for flight performance*	*Importance for knockdown time*
Sex	0.49	1.00
Oxygen concentration during development	0.67	—
Oxygen concentration during testing	0.36	0.05
Sex ∙ test oxygen	—	0.04
Temperature during testing	1.00	—
Developmental oxygen ∙ test oxygen	0.17	—
Test oxygen ∙ temperature	0.15	—
Developmental oxygen ∙ temperature	0.05	—
Developmental oxygen ∙ test oxygen ∙ temperature	< 0.01	—

**Fig 2:**
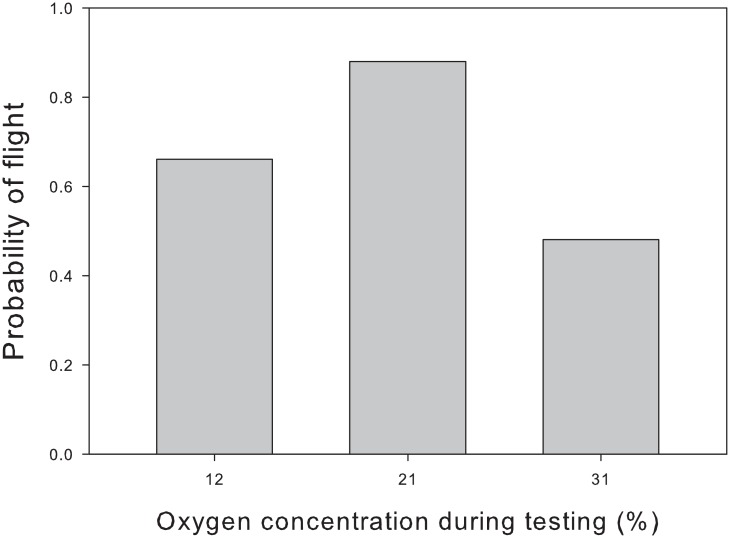
At 25°C, flies raised at normoxia performed best when tested at normoxia. The most likely probability of flight under each condition was computed by multimodel averaging. Fifty flies were tested at each concentration of oxygen.

Flight depended more on oxygen concentration during development than on oxygen concentration during testing (Table 3). At all temperatures, flies that developed at higher concentrations of oxygen were more likely to fly (Fig 1). A weak but interesting interaction emerged when flies were tested at 39°C. Flies that developed at normoxia or hyperoxia flew more often when tested at higher concentrations of oxygen. By contrast, flies that developed at hypoxia were more likely to fly when tested at lower concentrations of oxygen. At 37°C, flies from all developmental treatments flew most often when tested at normoxia, although the probability of flight exceeded 75% in all groups. These interactive effects were either relatively unimportant or too variable to detect with our samples, as evidenced by the low likelihood of models with these interactions (see Table 1). However, temperature likely influences the response to oxygen, because a much stronger effect of oxygen was observed when flies were tested at 25°C. At this temperature, both hypoxia and hyperoxia greatly reduced the probability of flight (Fig 2). In fact, flies at 25°C performed about as well or better at normoxia as did flies tested at 37°C.

**Fig 3:**
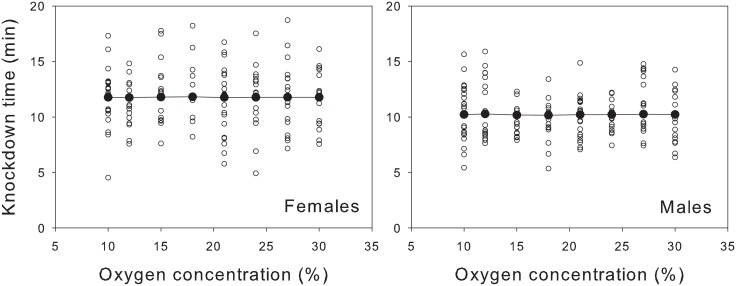
Oxygen during development had no effect on a fly’s ability to resist knockdown at 39.5°C. Large, solid symbols denote the most likely means estimated by multimodel averaging. Samples sizes were as follows: 23, 16, 17, 11, 21, 16, 17, and 17 females raised at 10%, 12%, 15%, 18%, 21%, 24%, 27%, and 30% oxygen, respectively; and 21, 18, 16, 13, 23, 16, 18, and 16 males raised at 10%, 12%, 15%, 18%, 21%, 24%, 27%, and 30% oxygen, respectively.

The heat resistance of resting flies was unrelated to their oxygen supply during development (Table 3). The most likely model of knockdown time included only the fixed effect of sex (see Table 2). This model was 95% likely to be the best model in the set, and was 22 times as likely as the second-ranked model (ΔAIC = 6.2). Although females took about 1.5 min longer to succumb to heat stress than males did, mean knockdown times for both sexes were virtually identical among groups that developed in different oxygen treatments (Fig 3).

## Discussion

Our results poorly support the oxygen limitation hypothesis. Raising the atmospheric concentration of oxygen from 12% to 31% had weak effects on flight performance at the stressful temperature of 39°C (see Fig 1). Although flies raised in normoxia and hyperoxia might have benefitted from hyperoxia, flies raised at hypoxia were less likely to fly at hyperoxia. Yet, we hesitate to generalize these patterns given how little variation was explained by this interaction (see Table 1). Furthermore, flies raised at hypoxia were unable to resist knockdown at 39.5°C any longer than were flies raised at hyperoxia (see Fig 3). Thus, we detected little or no evidence that heat tolerance acclimated to oxygen supply, regardless of whether heat tolerance was measured during activity or at rest. This result agrees with those of previous studies in which the lethal temperature of resting insects were unrelated to oxygen supply [8, 16, 40–42].

In general, developing at a higher level of oxygen conferred a major benefit to aerobic peformance during adulthood, which has been observed rarely in insects [15, 43]. At both 37° and 39°C, flies raised at hyperoxia were most likely to fly when tested at any concentration of oxygen. Interestingly, this developmental acclimation to hyperoxia imposed no loss of performance at hypoxia, which we expected as a tradeoff. This pattern accords with recent evidence that oxygen does not affect critical pO_2_ or tracheal conductance of flies [44]. Flies raised at hyperoxia could have reduced their investment in the tracheal system to such a degree that they were unable to deliver sufficient oxygen under hypoxia; if so, these flies would have performed worse than other flies when tested at hypoxia [24–27, 45]. Instead, these flies performed better than other flies at all oxygen concentrations. This greater performance could could have been a simple benefit of enhanced growth or reduced stress during development. In previous experiments, flies developing at hyperoxia reached a larger size at maturity than those developing at normoxia or hypoxia [24, 27, 46–49]. If flies were larger in hyperoxia, their size might have imparted an advantage during flight. Alternatively, or in conjunction with this hypothesis, by reducing the need to invest in an energetically costly tracheal system, these insects could increase their investment in flight muscle mass and other relevant tissues [28, 50]. These advantages could have resulted in a greater ratio of flight muscle to body size, which tends to scale hypermetrically, enhancing power and agility [51–55].

Because flies desiccate more quickly at higher temperatures, we should consider whether the knockdown times in our experiment were influences by hydric stress more than thermal stress. The knockdown assay lasted about 11 min on average (Fig 3), during which flies were in sealed vials with a relative humidity equal to that of the room (50%-60%). Based on previous experiments, this degree of hydric stress seems too benign to cause a knockdown response. Individuals of *D*. *melanogaster* resisted desiccation at 25°C and 0% humidity for an average of 10 to 80 hours [56, 57]. More relevant to our study of heat stress, flies resisted desiccation at 37°C and 0% humidity for an average of 48–53 minutes [58]. Although the temperature in our experiment was slightly higher, a knockdown time of 11 min in an environment with much greater humidity seems likely too short to attribute to desiccation. Therefore, the pattern of knockdown time reflects heat stress rather than hydric stress.

At lower temperatures, we oberserved a clear disadvantage to flying at either hypoxia or hyperoxia. We were surprised by this result, having hypothesized that hyperoxia would enhance aerobic metabolism during activity. Although we can only speculate about the cause of this pattern, flies at hyperoxia might have sacrificed their potential for aerobic metabolism to limit the production of reactive oxygen species. During a brief exposure to hyperoxia, as in our experiment, an insect could either reduce its exposure to reactive oxygen species or suffer damage to cells [15, 59–61]. In response to hyperoxia, some species of insects are known to close their spiracles [62–64]. In fact, researchers have argued that the respiratory patterns of insects evolved to avoid oxidative damage [65]. Contrary to this hypothesis, the oxygen level in the tracheal system of locusts directly matched that of the ambient air when exposed to hyperoxia [66]. Although this hypothesis remains unresolved, the mechanism that we propose would reduce flight performance by limiting the oxygen supply to cells despite the abundance of oxygen in the environment. This hypothetical mechanism has the advantage of fitting observations at higher temperatures as well. As body temperature increased, the demand for ATP might have increased to the point that only a tiny fraction of oxygen was reduced to form superoxide radicals instead of water [67, 68]. In line with this reasoning, the difference between performance at normoxia and performance at hyperoxia depended on the temperature: normoxia conferred a large advantage at 25°C, a mild advantage at 37°C, and a slight disadvantage at 39° or 41°C. If our hypothesis holds, a hyperoxic environment would impose a cost when flies demand less oxygen.

We have only begun to explore the interaction between heat and oxygen stresses when animals engage in energetically demanding yet ecologically relevant activities. Additional experiments are needed to support or refute the idea that oxygen can limit heat tolerance in terrestrial animals [9]. Although research has focused on a few species, which often reside in oxygen-rich environments, many animals live in soils that become hypoxic [69]. Some insects pass through larval stages that experience periods of hypoxia in rotting fruit, meat, or feces [70]. Other insects pass through aquatic stages before becoming terrestrial adults. The potential for oxygen levels during these stages to influence tolerance to hypoxia or heat at later stages remains largely unexplored. Future research should focus on heat tolerance during aerobically challenging activities at the oxygen levels encountered at specific stages of the life cycle.
